# P-852. Not High Enough? Treatment Outcome Comparing High Dose Daptomycin vs. Linezolid in VRE Bacteremia: Implications of Daptomycin PD Target

**DOI:** 10.1093/ofid/ofae631.1044

**Published:** 2025-01-29

**Authors:** Liang-En Hwang, Yu-Chung Chuang, Yee-Chun Chen, Jann-Tay Wang, Shan-Chwen Chang

**Affiliations:** National Taiwan University Hospital, Banciao, New Taipei, Taiwan (Republic of China); National Taiwan University Hospital and National Taiwan University College of Medicine, Taipei, Taipei, Taiwan; National Taiwan University Hospital, Banciao, New Taipei, Taiwan (Republic of China); National Taiwan University Hospital, Banciao, New Taipei, Taiwan (Republic of China); National Taiwan University Hospital, Banciao, New Taipei, Taiwan (Republic of China)

## Abstract

**Background:**

The best treatment for vancomycin-resistant enterococcus (VRE) bloodstream infection (BSI) remains controversial. In 2020, the CLSI revised the daptomycin breakpoints for *E. faecium* and recommended a high-dose daptomycin of 8-12 mg/kg/day. However, the effectiveness of high-dose daptomycin versus linezolid remains unanswered. The most effective PD parameter for daptomycin’s effectiveness is the free drug area under the curve to minimum inhibitory concentration ratio (fAUC/MIC). This study compares the clinical outcomes of VRE BSI between patients treated with linezolid and daptomycin within recommended dose. Additionally, we examined whether achieving the fAUC/MIC target influences the outcomes between these two groups.

**Figure 1.**
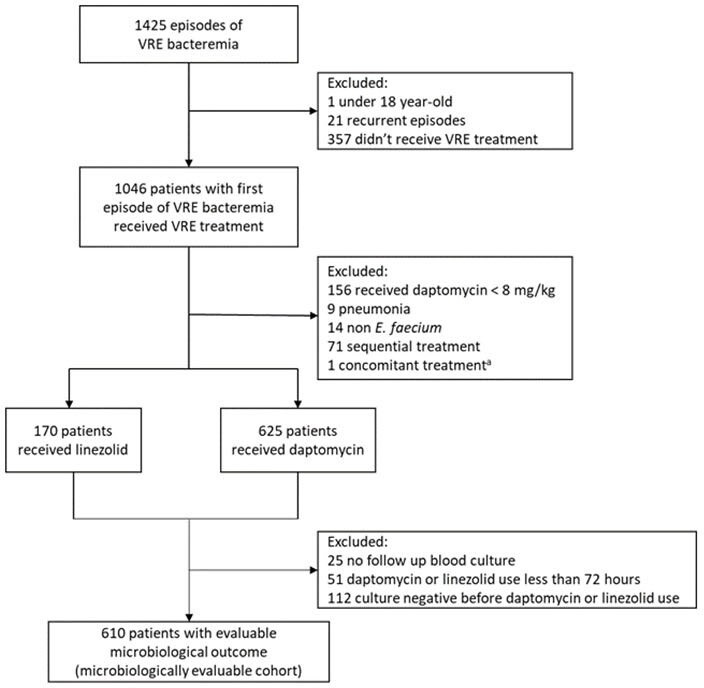
Flow diagram illustrating the selection of patients for the study.

**Methods:**

An observational cohort study was conducted within National Taiwan University Hospital and its branches. From January 2010 to December 2021, patients treated with more than 8 mg/kg of daptomycin or linezolid for VRE BSI were included. The estimated fAUC was calculated, and the fAUC/MIC target was chosen based on the MIC determined using broth microdilution. The primary outcome was in-hospital mortality.

**Table 1.**
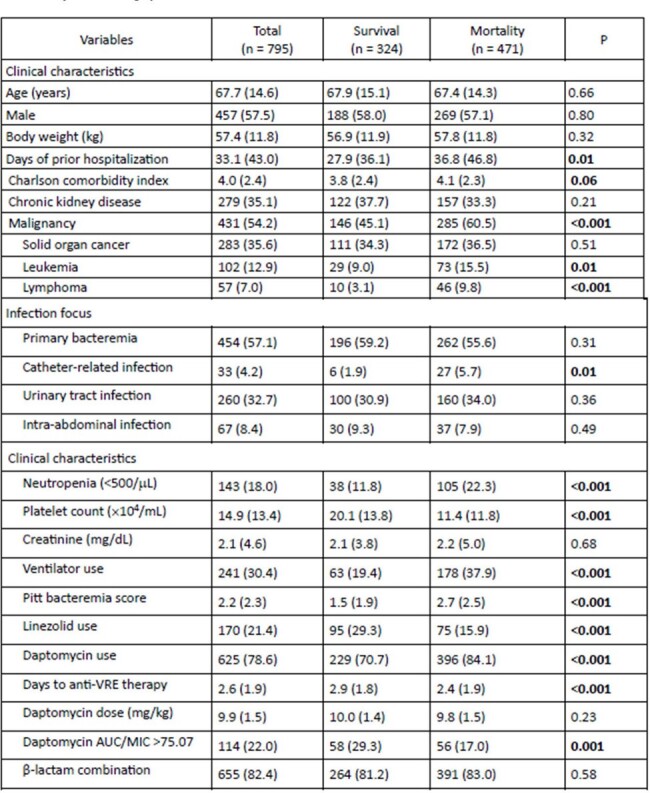
Demographics, clinical characteristics and univariable logistic regression analysis of mortality among patients with VRE BSI

**Results:**

There were 1425 VRE BSI episodes, and 795 met inclusion criteria. The mean age was 67.7 years (SD 14.6), with 57.5% males. The mortality was 59.3%. Of 625 daptomycin-treated patients, 528 had estimated fAUC/MIC available. 114 had estimated fAUC/MIC above target. Mortality was 49.1% for daptomycin-treated patients with the PD above target, 66.2% for those below it, and 44.1% for linezolid-treated patients. Multivariable logistic regression showed Charlson comorbidity index (aOR 1.90, P=0.05), platelet count (aOR 0.96, P< 0.001), and Pitt bacteremia score (aOR 1.31, P< 0.001) to be associated with mortality. Daptomycin below PD target was associated with worse outcome than linezolid (aOR 2.52, P< 0.001), while daptomycin above PD target was not (aOR 1.00, P=0.99).

**Figure 2.**
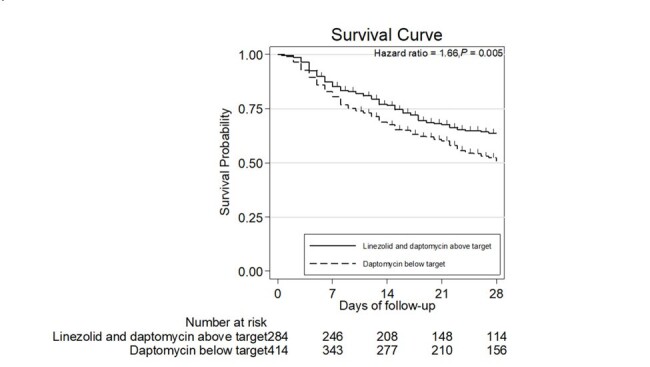
Comparison of Kaplan–Meier survival curves between daptomycin and linezolid in patients with VRE BSI

**Conclusion:**

Our findings suggest that daptomycin, even within the recommended dosage range, leads to poorer outcomes when it does not reach the estimated PD target compared to linezolid in treating VRE BSI. However, the outcomes are comparable with linezolid when PD target is reached.

**Table 2.**
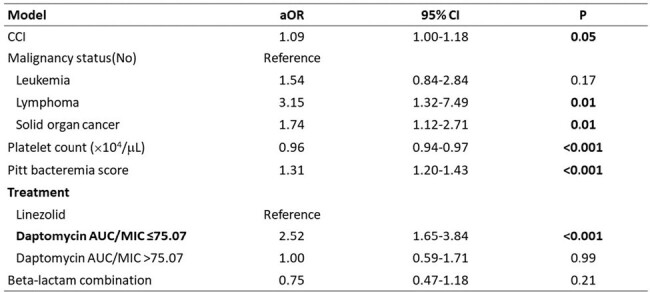
Multivariate logistic regression analysis of the factors associated with in-hospital mortality (N=695)

**Disclosures:**

**All Authors**: No reported disclosures

